# Epidemiologic Principles and Food Safety

**DOI:** 10.3201/eid1409.080585

**Published:** 2008-09

**Authors:** Timothy Jones

**Affiliations:** Author affiliation: Tennessee Department of Health, Nashville, Tennessee, USA

This text is an ambitious overview of the wide field of public health and food safety. Topics include foodborne pathogens, public health surveillance, basic epidemiologic principles, study design and outbreak management, food production, and food safety regulation. A substantial amount of attention has focused recently on the complexity of the food safety system in the United States, which includes a huge number of agencies, sometimes overlapping programs, databases and legal authority, and the inevitable consequence of inefficient and confusing communication. In light of these circumstances, this text provides a timely overview of the system that will be useful even to those very familiar with a particular aspect of the food safety continuum.

The descriptions of existing surveillance systems, sources of food consumption data, the federal regulatory system approach to risk assessment, food production and controls, and the regulatory environment are particularly clear, useful, and timely descriptions of sometimes vexingly complex systems. Clearly, such a breadth of topics cannot be addressed in great detail in a single text, but many of the subjects are seldom summarized so cogently, and readers interested in exploring particular topics will find this text provides a nice foundation for delving more deeply into the literature. This book provides something for everyone—an excellent overview for students, epidemiologists, regulators, academicians, industry representatives, and others interested in a broad survey of the field.

